# Google is goodish: An information literacy course designed to teach users why Google may not always be the best place to search for evidence

**DOI:** 10.1111/hir.12401

**Published:** 2021-09-20

**Authors:** Patricia Lacey

**Affiliations:** ^1^ Knowledge and Library Services Public Health England Birmingham UK

**Keywords:** education and training, health information needs, information literacy, information seeking behaviour, information skills, literature searching, public health, teaching

## Abstract

This article describes a course that was developed in response to health sector and local authority workers being reliant on Google and using it for their information needs regardless of whether it was the best place to search. The methodology for developing and structuring the course is explored, including details of the content included. The author concludes by asserting that teaching users about the effective use of Google is an important part of user education. D.I.

## BACKGROUND

As librarians and evidence specialists, we are well placed, as we always have been, to help people navigate to good‐quality resources and evidence. As the volume of published material continues to grow exponentially, this is becoming increasingly important. In May 2019, Google handled 92.04% of all web searches worldwide (Huisman et al., 2019). Google states that its mission is to organise the world's information and make it universally accessible and useful. It seeks to develop the perfect search engine *(*Carr, 2008 *)*. Google may find the answer but how trustworthy is it? Has it found everything? What did it miss? How systematic was the search? Searching Google for an answer is only part of the equation, and the other more important part is having the ability to recognise good‐quality information from reliable sources. As educators teaching reference and information searching skills, we face an uphill battle in prying Google's hammer out of students’ hands to help them learn other search tools (Ebrahim & Mon, 2011). In addition, most adults believe that search engines are fair and unbiased and often rely on ranking of search results for a credibility and selection making judgment (Gao & Shah, 2020).

There are many studies comparing Google Scholar and database searching, but there is a lack of research on why or how people use Google to search for evidence. There are, however, many anecdotal tales of librarians being told that Google will find the answer.

This article will discuss how a course demystifying Google was developed and run in order to help people who use Google working in the health and public sectors gain a better understanding of the search engine and the pitfalls associated with using it as a place to look for reliable evidence. The course is aimed at anyone who uses Google to search for evidence and anyone who would like more information about the risks of using Google.

## OBJECTIVES

The course was developed in response to post‐training feedback following literature search training that indicated those working in the health sector and Local Authorities found database searching difficult as they were reliant on using Google for all their information needs, including types of evidence. The Internet has no publishing filter and no mechanism for checking web pages; it can be prone to biases and gamification, and it can be manipulated. Although it can be used as a resource for finding evidence, it should not be the only resource used. After evaluation of the comments, we decided to design a course to develop users understanding of not relying on Google for literature searching.

The aim of the course was to provide an overview on why Google may not always be the best place to search for evidence covering eight main objectives: some common myths about Google, how search results are organised and presented (bias and search bubbles), fake websites, AstroTurf (definition below), fact or opinion, problems with Google scholar, tips for searching Google (when you really must) and how and where to fact check. Upon successful completion of the training, it was hoped that participants would understand some of the problems with using Google to conduct an evidence search, recognise fake websites and how to evaluate webpages in general, have an awareness of AstroTurf and knowledge that not all websites are created equal, and an understanding of some of the issues related to using Google Scholar.

## METHOD

### Course development

A prior course on searching Google was used as a foundation, and more research was then conducted on YouTube and Ted Talks to gather further insights and evidence for the course. A slide deck was produced, and more content added following attendance at a conference on fake news in particular a case study on the John Lott scandal (Blackwell, 2018).

The course was designed as a presentation to be run either face to face or virtually. The intention was for the session to be informative but light‐hearted. It was important that it was pitched correctly to enlighten but not alienate attendees. Rather than telling users ‘what you are doing is wrong’, the intention was to introduce topics that would enable them to see Google searching in a different light.

Ted Talks and YouTube clips were incorporated into the training because research shows that watching content can improve one's ability to remember concepts and details, and it is thought that viewers retain 95% of a message when they watch it in a video compared to 10% when reading it in text (Young, 2016). In addition, Stories and storytelling activities are excellent tools for increasing retention in training (Richter & Koppett, 2000).

### Course structure

There are seven main sections to the course:

1. Common myths about Google
2. Search results
3. Fake websites
4. Problems with Google scholar
5. Google searching
6. Other forms of ‘fake’ information
7. How to fact check

Section one covers common myths about Google including the following: the best information is found in the first ten results (not always), it is the only search engine available (it is not), you are certain to get the best, unbiased search result, or are you? Link to a Ted Talk on moral bias in search results. The reason for this session is to break down the common myths of using Google at the start. It is also important to highlight that the results you are seeing are not without biases, and there should be an awareness of this if you are going to search Google for information.

Section two looks at filter bubbles and links to a Ted Talk on this topic. This section also covers what can change your search results, for example the type of device used for the search, search history, geographic location, what type of browser you are using, it finishes with a closer look at Googles ‘feature snippet’. This is included to demonstrate that the results of a search can change by using either a different device or filter bubbles, and it is important to highlight that results are not static but fluid depending on a number of factors.

Section three uses the Pacific Northwest tree octopus as an example of a fake website. It then discusses Astro Turf. AstroTurf is when political, corporate, or other special interests disguise themselves and publish blogs, start Facebook and Twitter accounts, publish ads, letters to the editor, or simply post‐comments online to try to fool you into thinking it is an independent or grassroots movement is speaking. (Attkisson, 2015), you could think of it as manufacturing influence. This section includes a Ted Talk on AstroTurf and manipulation of media messages. To highlight the sophistication of fake websites, a quiz is included. Screen shots of fake and real websites are flashed up on screen and in response participants are asked to hold up either a ‘fake’ or ‘real’ card. Websites were chosen from a fake website blog (Bradley, 2011). If delivering the session virtually, mentimeter or other interactive presentation software can be used instead of cards. This section concludes with a YouTube clip on how to tell the difference between fact and opinions. The aim of introducing fake and real websites was to demonstrate how easy it is to be fooled, that not everything on the web is trustworthy and credible and that you need to be more cautious about the results you find. It is also important to highlight that the Internet can be manipulated in order to push a view or agenda.

Section four covers problems with Google Scholar and takes a closer look at what Google Scholar covers in its search, how is Google defining this? What do they search? Is there a bias towards what is searched? This is explored further by providing a search comparison between Google Scholar and Medline. This section demonstrates that searching Google Scholar is not the same as searching databases and to highlight what can be missed. It is also important to talk about the quality of the research and where this research comes from.

Section five looks at Google searching and demonstrates how searching for a topic can be phrased in several different ways. It also includes general search tips on how to search Google effectively. This was added in response to feedback that it would be useful to include some searching tips into the course. In terms of searching, it is important to highlight that a search question can be articulated in many ways and to highlight what can be missed as a result of this. It is impossible to do a systematic search for evidence using Google.

Section six covers other forms of ‘fake’ information such as fake videos, conspiracy theories, pseudoscience and alternative world views. This section can be included or excluded depending on time. Fake information is increasingly found in all forms, so it was important to include some information on this and provide users with some tips in how to recognise it.

Section seven goes through how to fact check websites. This was included in the course, to introduce users to appraising webpages for themselves.

### Format

The format of the training is flexible, so that it can be delivered successfully face to face or virtually, lasting an hour and half. Due to the pandemic, it has increasingly been delivered online using Microsoft Teams. The benefits of a virtual course include no travel or accommodation costs and no limits on the number of participants. The disadvantages are that it is more difficult to gauge audience reaction, and there is less participant interaction.

It is worth noting that the embedded videos means that the file size of the presentation is large, which makes it difficult to share or send via email. One solution to this has been to remove the video clips but add the link to the Ted Talks when sharing. There was also an issue with the video clips not playing over Skype, but this has since been solved with the move to Microsoft Teams.

## EVALUATION

The number of attendees varied from session to session ranging from 10 to 27. Attendees were from internal and external organisations and a range of backgrounds, with analysts, public health professionals, staff from local authorities and librarians making up the main groups.

In some cases, the session formed part of a larger training day. This is beneficial because those who would not have necessarily thought about undertaking the training themselves are targeted. The types of people motivated to undertake the training on their own tend to be either librarians with an interest in this area or those who are more proactive with a research background. Immediate feedback has been positive with attendees saying it had taught them something that they previously had not known; written feedback included the comment that the top take away was ‘do not Google’. Feedback from librarians has also been positive with many saying they now have some ammunition to use when they encounter comments such as ‘I’ll just Google it’ or ‘why do we need you or libraries when we have Google?’.

Further analysis would be useful to see whether attendees used databases after the course, and whether they were more effective in using Google and more selective in the results they used.

The advice to anyone considering running a course like this would be to try and find your key promoters/key users within the organisation and offer to run it as part of their larger training events. Now, it is also a really good time to push this agenda as there is increasing awareness about fake news and an abundance of misinformation and disinformation. People do not always make the connection that using Google means they are more likely to come across inaccurate information.

### Future developments

Google is constantly making changes to its algorithms, and as a result, the course should be checked regularly to make sure the information is still correct. News sources are currently scanned daily for any new developments, and any potential material is stored in a PowerPoint presentation for further investigation and future additions. This needs to be systematised, and there are plans to set up alerts and table of contents on relevant platforms, blogs and journals to keep abreast of the latest developments.

The content of the training is not static but evolving; material is added and taken away as the topic narrative and the discourse changes. The questions and feedback from attendees are used to inform the training, for example a few participants asked where the fake websites were from so the links to these will be added to the slides. This would also enable participants to be able to check the sites in their own time.

Although the initial decision to not teach users on how to search Google was made, in part because it contradicts the themes of the training, on reflection, there may be some values into expanding the section on Google search tips to include Boolean logic and some search examples. By using a search tool that students are already familiar with, we can introduce concepts and techniques of more advanced information searching which also helps users to remember the skills when using them in connection with other online subscription databases (Ebrahim & Mon, 2011).

Since the initial development of the Google is Goodish course, fake news and infodemics have come to the fore as pertinent issues and it was felt that these issues needed to be addressed separately. In February 2020, the World Health Organisation warned that, alongside the outbreak of COVID‐19, the world faced an ‘infodemic’, an unprecedented overabundance of information both accurate and false that prevented people from accessing authoritative, reliable guidance about the virus. (Editorial, 2020). There is increasing user demand to learn more about misinformation and disinformation. However, due to time constraints it is not possible to fully address these issues within the training, and therefore, a separate course on fake news has been developed and run as a course on its own.

There are no plans to produce this as an e‐learning course, but there are plans to record a version and host this on the PHE Knowledge and Library Services WordPress pages for external and internal audiences.

## CONCLUSION

Running the course has increased awareness of the risks of using Google to search for evidence. It has encouraged people to learn how to search effectively by using the databases, and in doing so, they have gained a better understanding of what reliable evidence is and where to find it.

In the current climate, good information literacy skills have never been so important. Library efforts must be geared towards discerning what authentic and dependable sources of information are (Durodolu & Ibenne, 2020), and although this was written about fake news, it applies equally here as Google is a source of information. As librarians and information professionals, we deal with information resources as part of our profession and, as a result, have invaluable skills at recognising reliable information from ‘authentic and dependable sources’; others without this skill may find it harder and using Google to search for evidence adds an additional level of complexity. Most of us offer some form of information literacy training (including digital literacy) as part of our offer to users, teaching users about some of the pitfalls of only relying on Google and other search engines for evidence can play an important role in user education.

The author is keen to communicate and collaborate with other librarians, and information specialist who also have a mutual interest in this topic.
